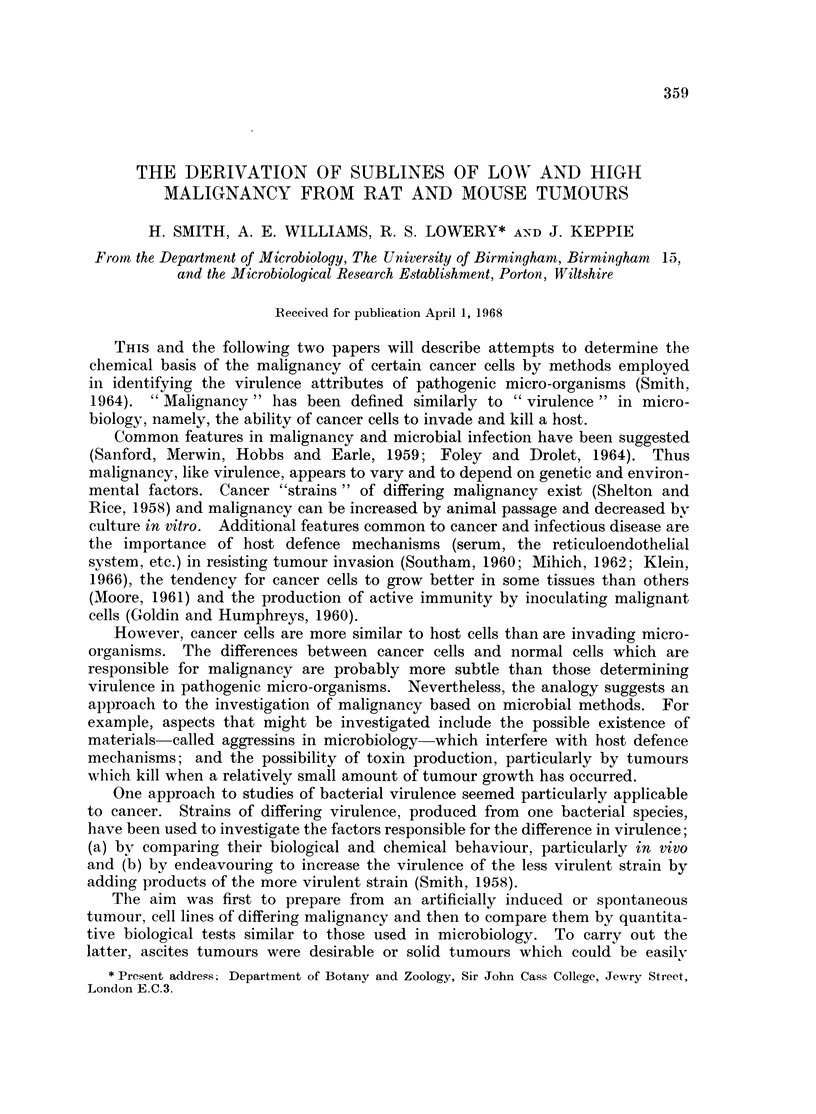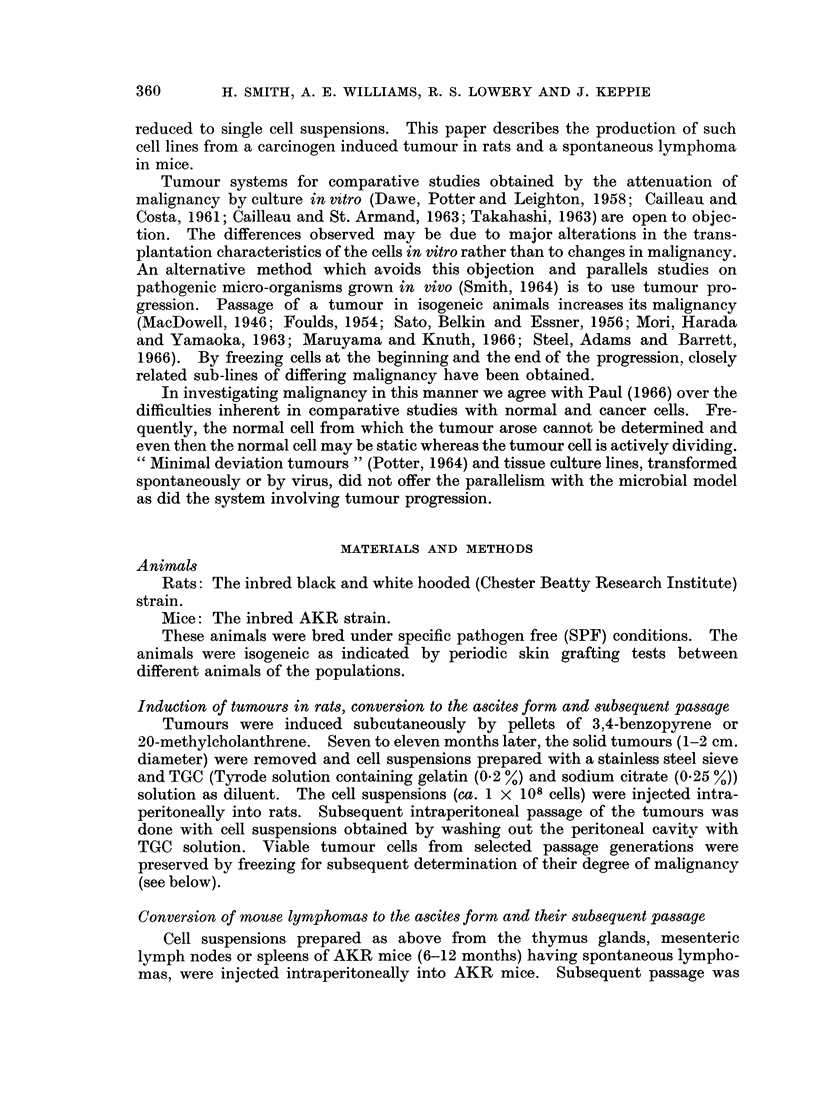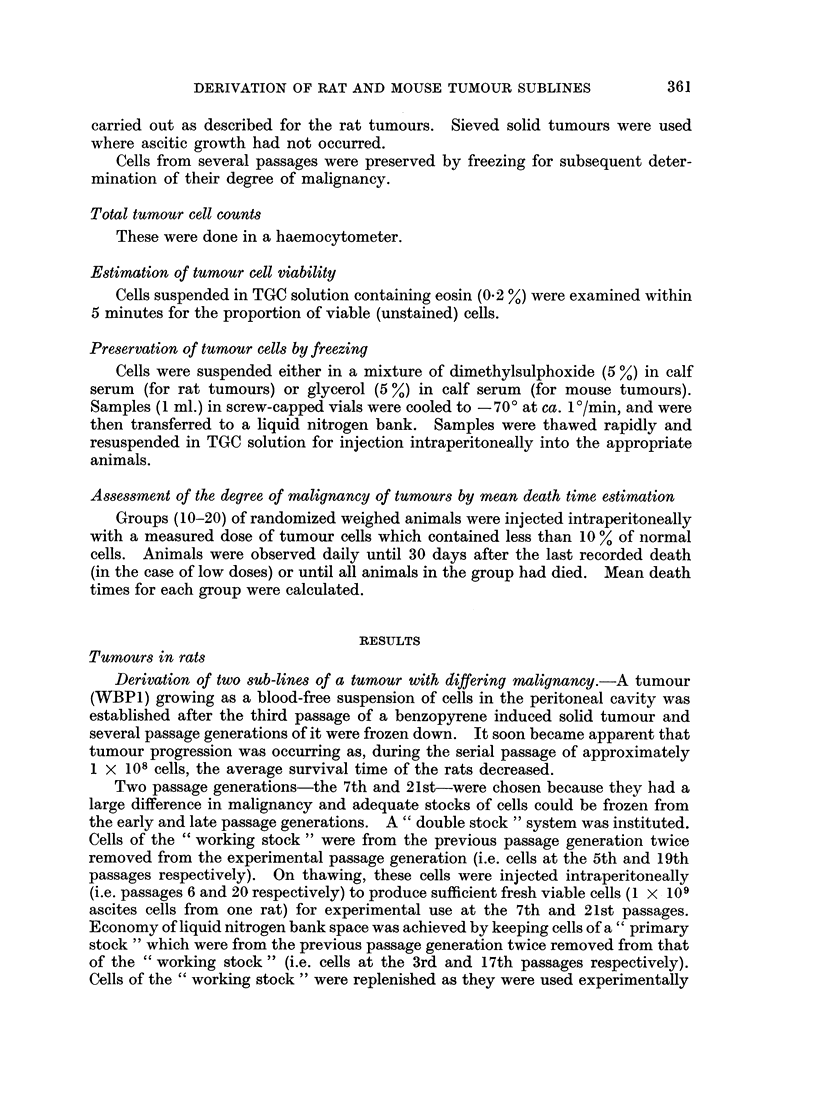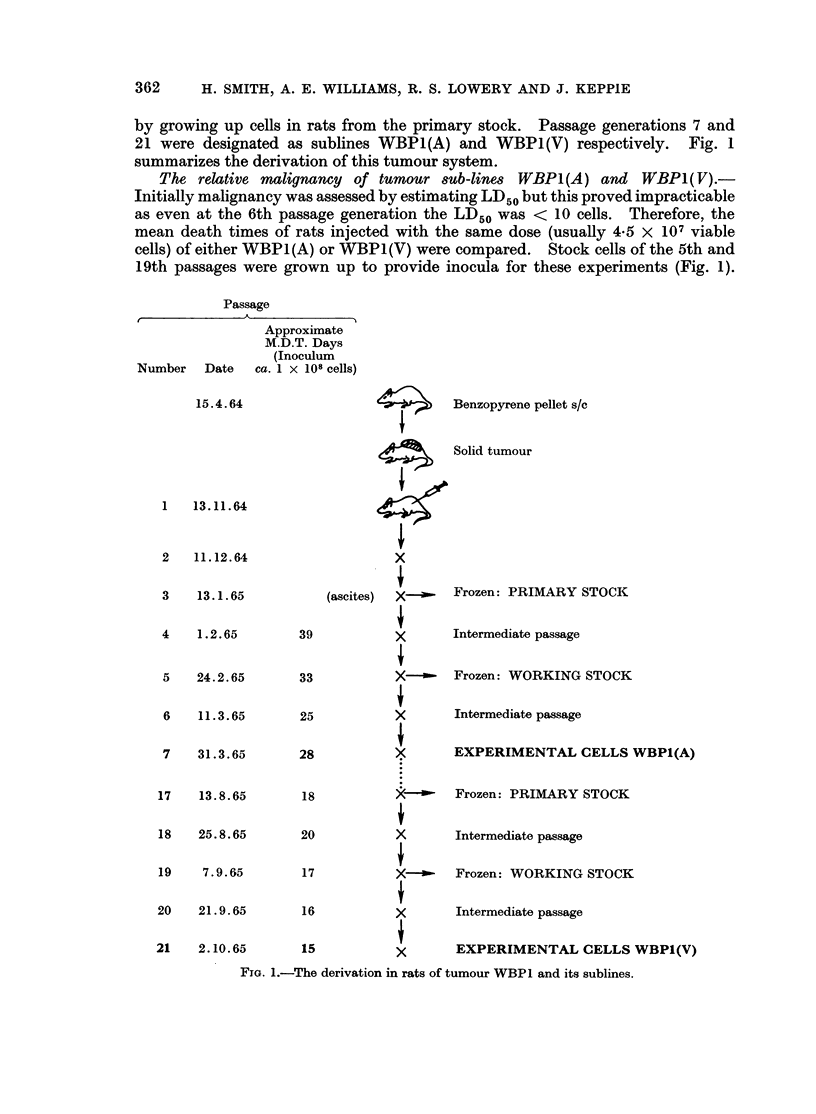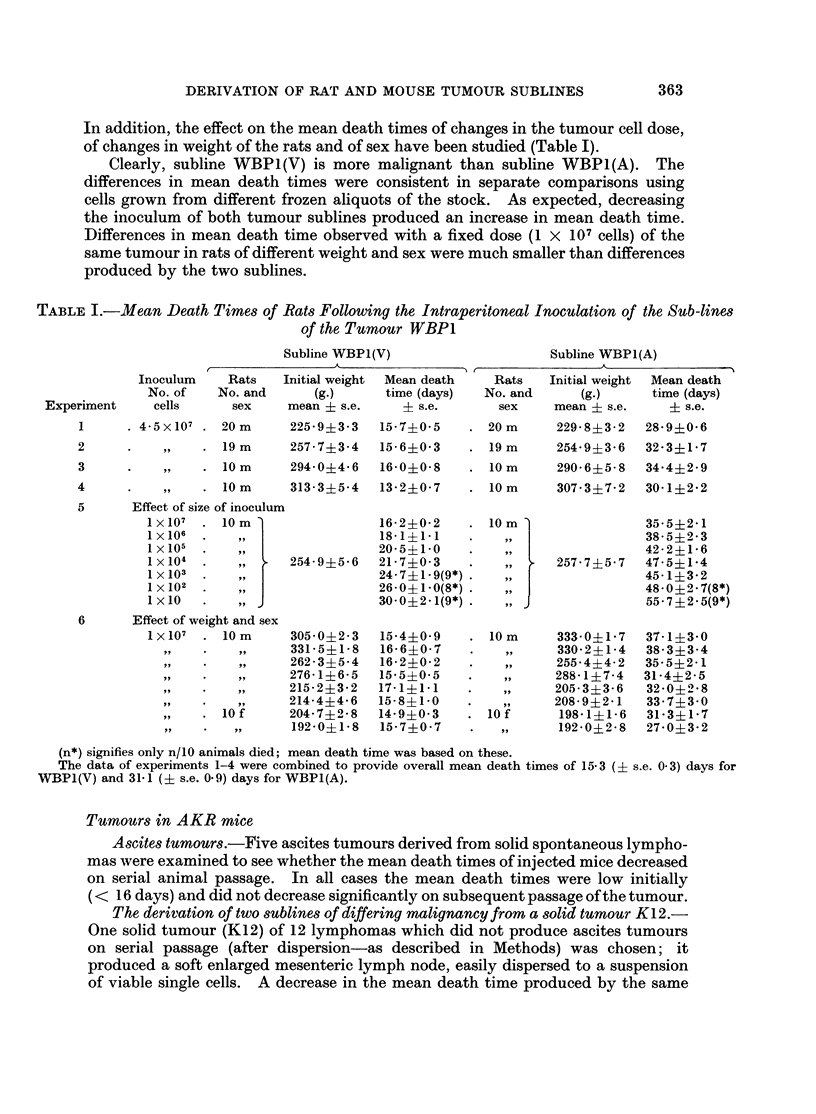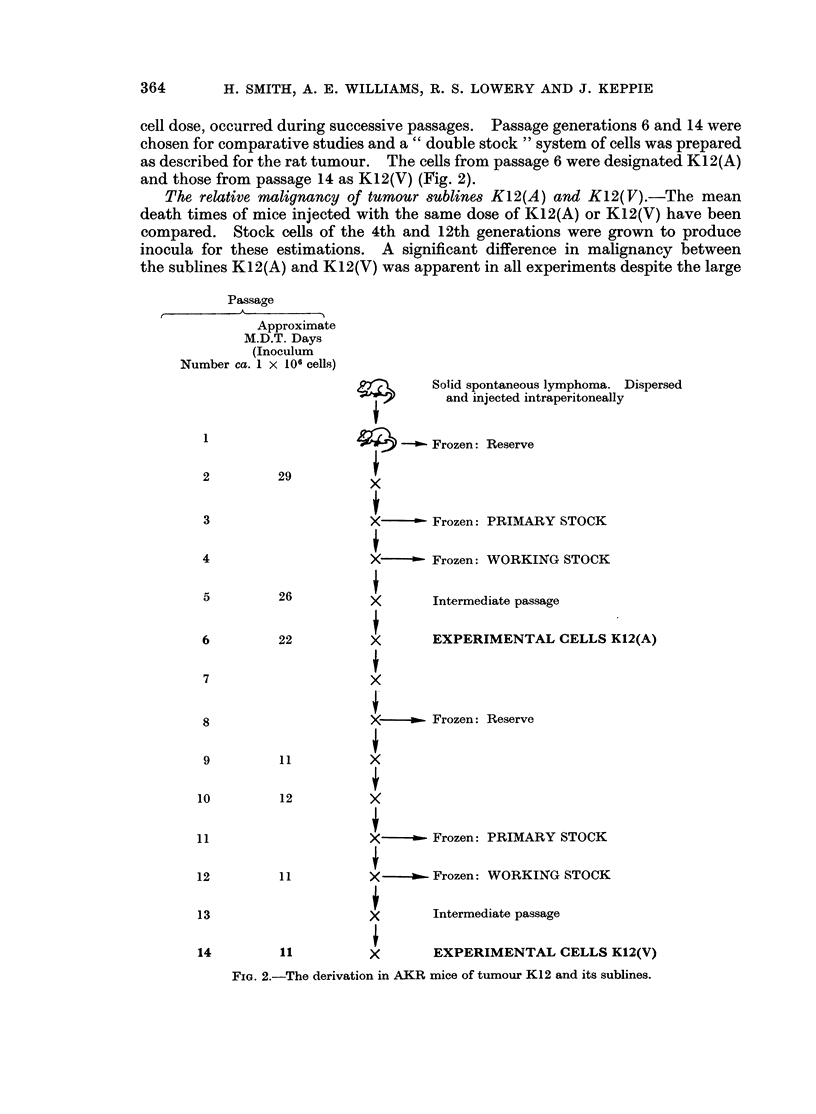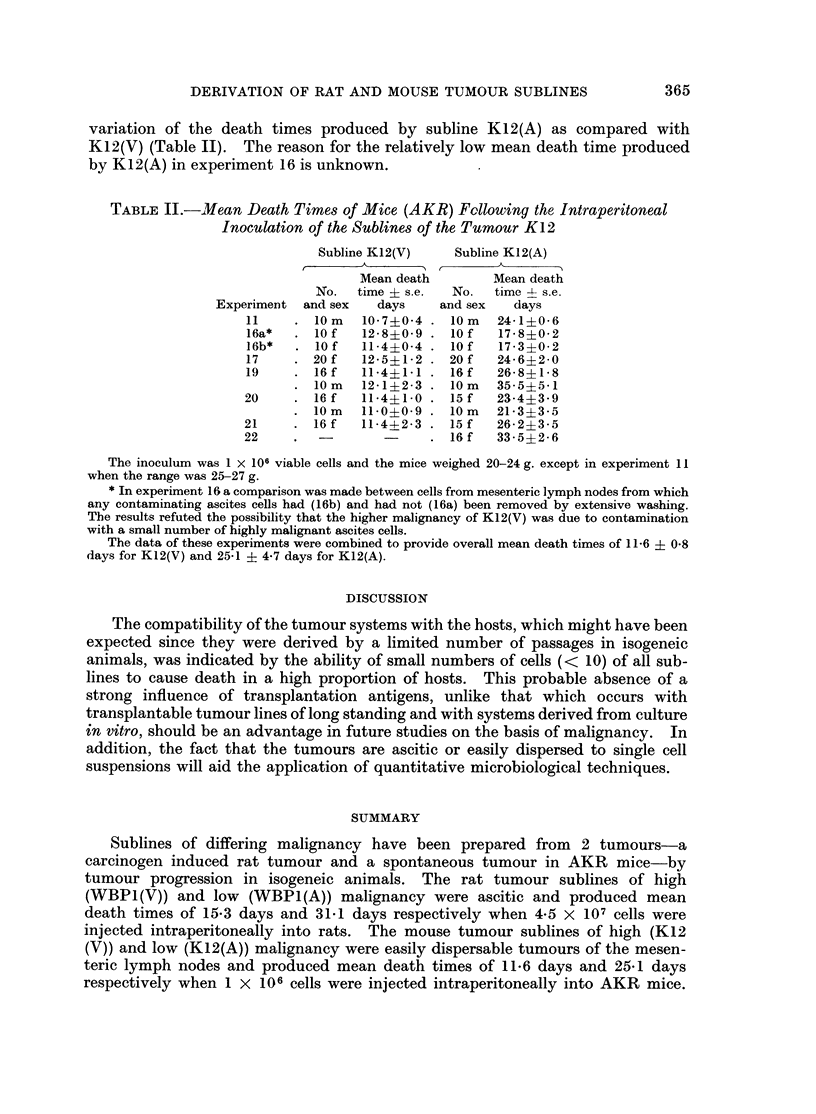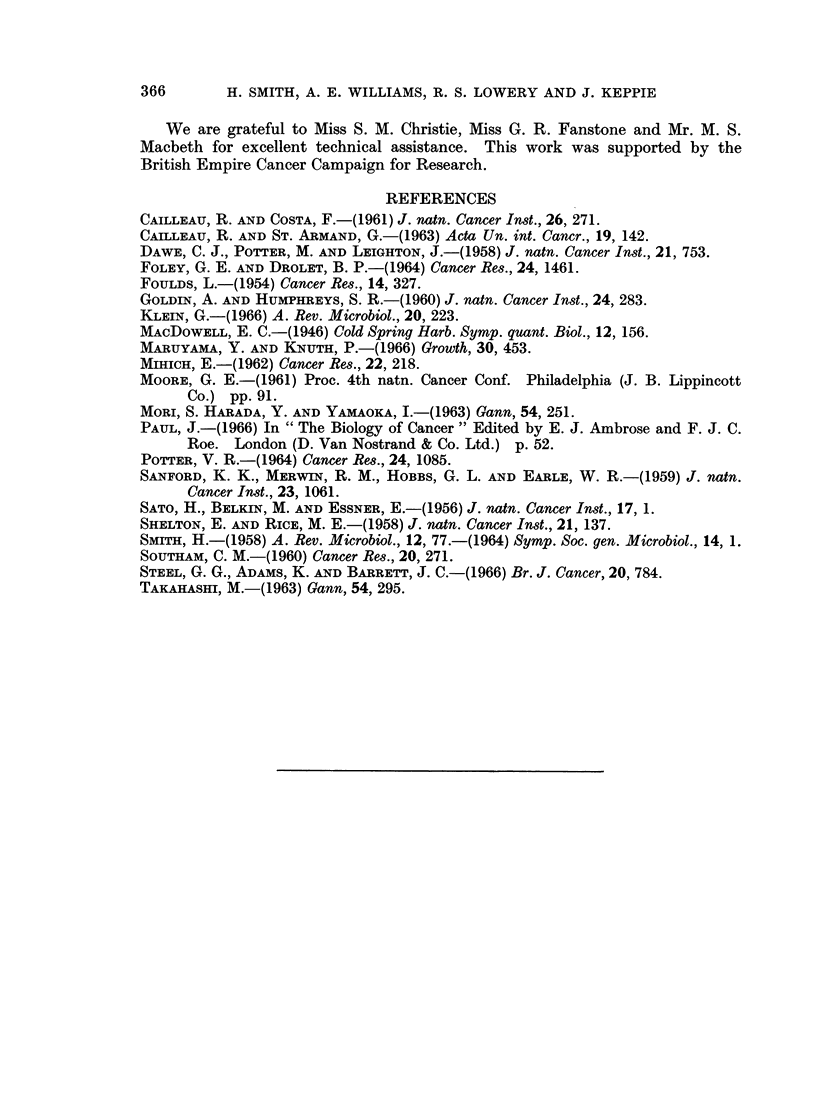# The derivation of sublines of low and high malignancy from rat and mouse tumours.

**DOI:** 10.1038/bjc.1968.44

**Published:** 1968-06

**Authors:** H. Smith, A. E. Williams, R. S. Lowery, J. Keppie


### THE DERIVATION OF SUBLINES OF LOW AND HIGH MALIGNANCY FROM RAT AND MOUSE TUMOURS

H. SMITH, A. E. WILLIAMS, R. S. LOWERY* AND J. KEPPIE

*From the Department of Microbiology, The University of Birmingham, Birmingham 15, and the Microbiological Research Establishment, Porton, Wiltshire*

Received for publication April 1, 1968

THIS and the following two papers will describe attempts to determine the chemical basis of the malignancy of certain cancer cells by methods employed in identifying the virulence attributes of pathogenic micro-organisms (Smith, 1964). " Malignancy " has been defined similarly to " virulence " in microbiology, namely, the ability of cancer cells to invade and kill a host.

Common features in malignancy and microbial infection have been suggested (Sanford, Merwin, Hobbs and Earle, 1959; Foley and Drolet, 1964). Thus malignancy, like virulence, appears to vary and to depend on genetic and environmental factors. Cancer "strains " of differing malignancy exist (Shelton and Rice, 1958) and malignancy can be increased by animal passage and decreased by culture *in vitro*. Additional features common to cancer and infectious disease are the importance of host defence mechanisms (serum, the reticuloendothelial system, etc.) in resisting tumour invasion (Southam, 1960; Mihich, 1962; Klein, 1966), the tendency for cancer cells to grow better in some tissues than others (Moore, 1961) and the production of active immunity by inoculating malignant cells (Goldin and Humphreys, 1960).

However, cancer cells are more similar to host cells than are invading micro-organisms. The differences between cancer cells and normal cells which are responsible for malignancy are probably more subtle than those determining virulence in pathogenic micro-organisms. Nevertheless, the analogy suggests an approach to the investigation of malignancy based on microbial methods. For example, aspects that might be investigated include the possible existence of materials—called aggressins in microbiology—which interfere with host defence mechanisms; and the possibility of toxin production, particularly by tumours which kill when a relatively small amount of tumour growth has occurred.

One approach to studies of bacterial virulence seemed particularly applicable to cancer. Strains of differing virulence, produced from one bacterial species, have been used to investigate the factors responsible for the difference in virulence; (a) by comparing their biological and chemical behaviour, particularly *in vivo* and (b) by endeavouring to increase the virulence of the less virulent strain by adding products of the more virulent strain (Smith, 1958).

The aim was first to prepare from an artificially induced or spontaneous tumour, cell lines of differing malignancy and then to compare them by quantitative biological tests similar to those used in microbiology. To carry out the latter, ascites tumours were desirable or solid tumours which could be easily

* Present address: Department of Botany and Zoology, Sir John Cass College, Jewry Street, London E.C.3.

reduced to single cell suspensions. This paper describes the production of such cell lines from a carcinogen induced tumour in rats and a spontaneous lymphoma in mice.

Tumour systems for comparative studies obtained by the attenuation of malignancy by culture *in vitro* (Dawe, Potter and Leighton, 1958; Cailleau and Costa, 1961; Cailleau and St. Armand, 1963; Takahashi, 1963) are open to objection. The differences observed may be due to major alterations in the transplantation characteristics of the cells *in vitro* rather than to changes in malignancy. An alternative method which avoids this objection and parallels studies on pathogenic micro-organisms grown *in vivo* (Smith, 1964) is to use tumour progression. Passage of a tumour in isogeneic animals increases its malignancy (MacDowell, 1946; Foulds, 1954; Sato, Belkin and Essner, 1956; Mori, Harada and Yamaoka, 1963; Maruyama and Knuth, 1966; Steel, Adams and Barrett, 1966). By freezing cells at the beginning and the end of the progression, closely related sub-lines of differing malignancy have been obtained.

In investigating malignancy in this manner we agree with Paul (1966) over the difficulties inherent in comparative studies with normal and cancer cells. Frequently, the normal cell from which the tumour arose cannot be determined and even then the normal cell may be static whereas the tumour cell is actively dividing. " Minimal deviation tumours " (Potter, 1964) and tissue culture lines, transformed spontaneously or by virus, did not offer the parallelism with the microbial model as did the system involving tumour progression.

### MATERIALS AND METHODS

*Animals*

Rats: The inbred black and white hooded (Chester Beatty Research Institute) strain.

Mice: The inbred AKR strain.

These animals were bred under specific pathogen free (SPF) conditions. The animals were isogeneic as indicated by periodic skin grafting tests between different animals of the populations.

*Induction of tumours in rats, conversion to the ascites form and subsequent passage*

Tumours were induced subcutaneously by pellets of 3,4-benzopyrene or 20-methylcholanthrene. Seven to eleven months later, the solid tumours (1–2 cm. diameter) were removed and cell suspensions prepared with a stainless steel sieve and TGC (Tyrode solution containing gelatin (0·2 %) and sodium citrate (0·25 %)) solution as diluent. The cell suspensions (*ca.* $1 \times 10^8$ cells) were injected intraperitoneally into rats. Subsequent intraperitoneal passage of the tumours was done with cell suspensions obtained by washing out the peritoneal cavity with TGC solution. Viable tumour cells from selected passage generations were preserved by freezing for subsequent determination of their degree of malignancy (see below).

*Conversion of mouse lymphomas to the ascites form and their subsequent passage*

Cell suspensions prepared as above from the thymus glands, mesenteric lymph nodes or spleens of AKR mice (6–12 months) having spontaneous lymphomas, were injected intraperitoneally into AKR mice. Subsequent passage was carried out as described for the rat tumours. Sieved solid tumours were used where ascitic growth had not occurred.

Cells from several passages were preserved by freezing for subsequent determination of their degree of malignancy.

*Total tumour cell counts*

These were done in a haemocytometer.

*Estimation of tumour cell viability*

Cells suspended in TGC solution containing eosin (0·2 %) were examined within 5 minutes for the proportion of viable (unstained) cells.

*Preservation of tumour cells by freezing*

Cells were suspended either in a mixture of dimethylsulphoxide (5 %) in calf serum (for rat tumours) or glycerol (5 %) in calf serum (for mouse tumours). Samples (1 ml.) in screw-capped vials were cooled to $-70°$ at *ca.* 1°/min, and were then transferred to a liquid nitrogen bank. Samples were thawed rapidly and resuspended in TGC solution for injection intraperitoneally into the appropriate animals.

*Assessment of the degree of malignancy of tumours by mean death time estimation*

Groups (10–20) of randomized weighed animals were injected intraperitoneally with a measured dose of tumour cells which contained less than 10 % of normal cells. Animals were observed daily until 30 days after the last recorded death (in the case of low doses) or until all animals in the group had died. Mean death times for each group were calculated.

### RESULTS

*Tumours in rats*

*Derivation of two sub-lines of a tumour with differing malignancy.*—A tumour (WBP1) growing as a blood-free suspension of cells in the peritoneal cavity was established after the third passage of a benzopyrene induced solid tumour and several passage generations of it were frozen down. It soon became apparent that tumour progression was occurring as, during the serial passage of approximately $1 \times 10^8$ cells, the average survival time of the rats decreased.

Two passage generations—the 7th and 21st—were chosen because they had a large difference in malignancy and adequate stocks of cells could be frozen from the early and late passage generations. A " double stock " system was instituted. Cells of the " working stock " were from the previous passage generation twice removed from the experimental passage generation (i.e. cells at the 5th and 19th passages respectively). On thawing, these cells were injected intraperitoneally (i.e. passages 6 and 20 respectively) to produce sufficient fresh viable cells ($1 \times 10^9$ ascites cells from one rat) for experimental use at the 7th and 21st passages. Economy of liquid nitrogen bank space was achieved by keeping cells of a " primary stock " which were from the previous passage generation twice removed from that of the " working stock " (i.e. cells at the 3rd and 17th passages respectively). Cells of the " working stock " were replenished as they were used experimentally by growing up cells in rats from the primary stock. Passage generations 7 and 21 were designated as sublines WBP1(A) and WBP1(V) respectively. Fig. 1 summarizes the derivation of this tumour system.

*The relative malignancy of tumour sub-lines WBP1(A) and WBP1(V).*—Initially malignancy was assessed by estimating $LD_{50}$ but this proved impracticable as even at the 6th passage generation the $LD_{50}$ was $< 10$ cells. Therefore, the mean death times of rats injected with the same dose (usually $4{\cdot}5 \times 10^7$ viable cells) of either WBP1(A) or WBP1(V) were compared. Stock cells of the 5th and 19th passages were grown up to provide inocula for these experiments (Fig. 1).

| Passage | | | |
|---|---|---|---|
| Number | Date | Approximate M.D.T. Days (Inoculum *ca.* $1 \times 10^8$ cells) | |
| | 15.4.64 | | Benzopyrene pellet s/c |
| | | | Solid tumour |
| 1 | 13.11.64 | | |
| 2 | 11.12.64 | | |
| 3 | 13.1.65 | (ascites) | Frozen: PRIMARY STOCK |
| 4 | 1.2.65 | 39 | Intermediate passage |
| 5 | 24.2.65 | 33 | Frozen: WORKING STOCK |
| 6 | 11.3.65 | 25 | Intermediate passage |
| **7** | 31.3.65 | **28** | **EXPERIMENTAL CELLS WBP1(A)** |
| 17 | 13.8.65 | 18 | Frozen: PRIMARY STOCK |
| 18 | 25.8.65 | 20 | Intermediate passage |
| 19 | 7.9.65 | 17 | Frozen: WORKING STOCK |
| 20 | 21.9.65 | 16 | Intermediate passage |
| **21** | 2.10.65 | **15** | **EXPERIMENTAL CELLS WBP1(V)** |

FIG. 1.—The derivation in rats of tumour WBP1 and its sublines.

In addition, the effect on the mean death times of changes in the tumour cell dose, of changes in weight of the rats and of sex have been studied (Table I).

Clearly, subline WBP1(V) is more malignant than subline WBP1(A). The differences in mean death times were consistent in separate comparisons using cells grown from different frozen aliquots of the stock. As expected, decreasing the inoculum of both tumour sublines produced an increase in mean death time. Differences in mean death time observed with a fixed dose ($1 \times 10^7$ cells) of the same tumour in rats of different weight and sex were much smaller than differences produced by the two sublines.

TABLE I.—*Mean Death Times of Rats Following the Intraperitoneal Inoculation of the Sub-lines of the Tumour WBP*1

| Experiment | Inoculum No. of cells | Subline WBP1(V) Rats No. and sex | Initial weight (g.) mean ± s.e. | Mean death time (days) ± s.e. | Subline WBP1(A) Rats No. and sex | Initial weight (g.) mean ± s.e. | Mean death time (days) ± s.e. |
|---|---|---|---|---|---|---|---|
| 1 | $4 \cdot 5 \times 10^7$ | 20 m | 225·9±3·3 | 15·7±0·5 | 20 m | 229·8±3·2 | 28·9±0·6 |
| 2 | ,, | 19 m | 257·7±3·4 | 15·6±0·3 | 19 m | 254·9±3·6 | 32·3±1·7 |
| 3 | ,, | 10 m | 294·0±4·6 | 16·0±0·8 | 10 m | 290·6±5·8 | 34·4±2·9 |
| 4 | ,, | 10 m | 313·3±5·4 | 13·2±0·7 | 10 m | 307·3±7·2 | 30·1±2·2 |
| 5 | Effect of size of inoculum | | | | | | |
| | $1 \times 10^7$ | 10 m | 254·9±5·6 | 16·2±0·2 | 10 m | 257·7±5·7 | 35·5±2·1 |
| | $1 \times 10^6$ | ,, | | 18·1±1·1 | ,, | | 38·5±2·3 |
| | $1 \times 10^5$ | ,, | | 20·5±1·0 | ,, | | 42·2±1·6 |
| | $1 \times 10^4$ | ,, | | 21·7±0·3 | ,, | | 47·5±1·4 |
| | $1 \times 10^3$ | ,, | | 24·7±1·9(9*) | ,, | | 45·1±3·2 |
| | $1 \times 10^2$ | ,, | | 26·0±1·0(8*) | ,, | | 48·0±2·7(8*) |
| | $1 \times 10$ | ,, | | 30·0±2·1(9*) | ,, | | 55·7±2·5(9*) |
| 6 | Effect of weight and sex | | | | | | |
| | $1 \times 10^7$ | 10 m | 305·0±2·3 | 15·4±0·9 | 10 m | 333·0±1·7 | 37·1±3·0 |
| | ,, | ,, | 331·5±1·8 | 16·6±0·7 | ,, | 330·2±1·4 | 38·3±3·4 |
| | ,, | ,, | 262·3±5·4 | 16·2±0·2 | ,, | 255·4±4·2 | 35·5±2·1 |
| | ,, | ,, | 276·1±6·5 | 15·5±0·5 | ,, | 288·1±7·4 | 31·4±2·5 |
| | ,, | ,, | 215·2±3·2 | 17·1±1·1 | ,, | 205·3±3·6 | 32·0±2·8 |
| | ,, | ,, | 214·4±4·6 | 15·8±1·0 | ,, | 208·9±2·1 | 33·7±3·0 |
| | ,, | 10 f | 204·7±2·8 | 14·9±0·3 | 10 f | 198·1±1·6 | 31·3±1·7 |
| | ,, | ,, | 192·0±1·8 | 15·7±0·7 | ,, | 192·0±2·8 | 27·0±3·2 |

(n*) signifies only n/10 animals died; mean death time was based on these.

The data of experiments 1–4 were combined to provide overall mean death times of 15·3 (± s.e. 0·3) days for WBP1(V) and 31·1 (± s.e. 0·9) days for WBP1(A).

### *Tumours in AKR mice*

*Ascites tumours.*—Five ascites tumours derived from solid spontaneous lymphomas were examined to see whether the mean death times of injected mice decreased on serial animal passage. In all cases the mean death times were low initially (< 16 days) and did not decrease significantly on subsequent passage of the tumour.

*The derivation of two sublines of differing malignancy from a solid tumour K*12.—One solid tumour (K12) of 12 lymphomas which did not produce ascites tumours on serial passage (after dispersion—as described in Methods) was chosen; it produced a soft enlarged mesenteric lymph node, easily dispersed to a suspension of viable single cells. A decrease in the mean death time produced by the same cell dose, occurred during successive passages. Passage generations 6 and 14 were chosen for comparative studies and a " double stock " system of cells was prepared as described for the rat tumour. The cells from passage 6 were designated K12(A) and those from passage 14 as K12(V) (Fig. 2).

*The relative malignancy of tumour sublines K*12(*A*) *and K*12(*V*).—The mean death times of mice injected with the same dose of K12(A) or K12(V) have been compared. Stock cells of the 4th and 12th generations were grown to produce inocula for these estimations. A significant difference in malignancy between the sublines K12(A) and K12(V) was apparent in all experiments despite the large

FIG. 2.—The derivation in AKR mice of tumour K12 and its sublines.

variation of the death times produced by subline K12(A) as compared with K12(V) (Table II). The reason for the relatively low mean death time produced by K12(A) in experiment 16 is unknown.

TABLE II.—*Mean Death Times of Mice (AKR) Following the Intraperitoneal Inoculation of the Sublines of the Tumour K*12

| | Subline K12(V) | | Subline K12(A) | |
|---|---|---|---|---|
| Experiment | No. and sex | Mean death time ± s.e. days | No. and sex | Mean death time ± s.e. days |
| 11 | 10 m | 10·7±0·4 | 10 m | 24·1±0·6 |
| 16a* | 10 f | 12·8±0·9 | 10 f | 17·8±0·2 |
| 16b* | 10 f | 11·4±0·4 | 10 f | 17·3±0·2 |
| 17 | 20 f | 12·5±1·2 | 20 f | 24·6±2·0 |
| 19 | 16 f | 11·4±1·1 | 16 f | 26·8±1·8 |
| | 10 m | 12·1±2·3 | 10 m | 35·5±5·1 |
| 20 | 16 f | 11·4±1·0 | 15 f | 23·4±3·9 |
| | 10 m | 11·0±0·9 | 10 m | 21·3±3·5 |
| 21 | 16 f | 11·4±2·3 | 15 f | 26·2±3·5 |
| 22 | — | — | 16 f | 33·5±2·6 |

The inoculum was $1 \times 10^6$ viable cells and the mice weighed 20–24 g. except in experiment 11 when the range was 25–27 g.

* In experiment 16 a comparison was made between cells from mesenteric lymph nodes from which any contaminating ascites cells had (16b) and had not (16a) been removed by extensive washing. The results refuted the possibility that the higher malignancy of K12(V) was due to contamination with a small number of highly malignant ascites cells.

The data of these experiments were combined to provide overall mean death times of 11·6 ± 0·8 days for K12(V) and 25·1 ± 4·7 days for K12(A).

### DISCUSSION

The compatibility of the tumour systems with the hosts, which might have been expected since they were derived by a limited number of passages in isogeneic animals, was indicated by the ability of small numbers of cells (< 10) of all sublines to cause death in a high proportion of hosts. This probable absence of a strong influence of transplantation antigens, unlike that which occurs with transplantable tumour lines of long standing and with systems derived from culture *in vitro*, should be an advantage in future studies on the basis of malignancy. In addition, the fact that the tumours are ascitic or easily dispersed to single cell suspensions will aid the application of quantitative microbiological techniques.

### SUMMARY

Sublines of differing malignancy have been prepared from 2 tumours—a carcinogen induced rat tumour and a spontaneous tumour in AKR mice—by tumour progression in isogeneic animals. The rat tumour sublines of high (WBP1(V)) and low (WBP1(A)) malignancy were ascitic and produced mean death times of 15·3 days and 31·1 days respectively when $4{\cdot}5 \times 10^7$ cells were injected intraperitoneally into rats. The mouse tumour sublines of high (K12 (V)) and low (K12(A)) malignancy were easily dispersable tumours of the mesenteric lymph nodes and produced mean death times of 11·6 days and 25·1 days respectively when $1 \times 10^6$ cells were injected intraperitoneally into AKR mice.

We are grateful to Miss S. M. Christie, Miss G. R. Fanstone and Mr. M. S. Macbeth for excellent technical assistance. This work was supported by the British Empire Cancer Campaign for Research.

### REFERENCES

Cailleau, R. and Costa, F.—(1961) *J. natn. Cancer Inst.*, **26**, 271.
Cailleau, R. and St. Armand, G.—(1963) *Acta Un. int. Cancr.*, **19**, 142.
Dawe, C. J., Potter, M. and Leighton, J.—(1958) *J. natn. Cancer Inst.*, **21**, 753.
Foley, G. E. and Drolet, B. P.—(1964) *Cancer Res.*, **24**, 1461.
Foulds, L.—(1954) *Cancer Res.*, **14**, 327.
Goldin, A. and Humphreys, S. R.—(1960) *J. natn. Cancer Inst.*, **24**, 283.
Klein, G.—(1966) *A. Rev. Microbiol.*, **20**, 223.
MacDowell, E. C.—(1946) *Cold Spring Harb. Symp. quant. Biol.*, **12**, 156.
Maruyama, Y. and Knuth, P.—(1966) *Growth*, **30**, 453.
Mihich, E.—(1962) *Cancer Res.*, **22**, 218.
Moore, G. E.—(1961) Proc. 4th natn. Cancer Conf. Philadelphia (J. B. Lippincott Co.) pp. 91.
Mori, S. Harada, Y. and Yamaoka, I.—(1963) *Gann*, **54**, 251.
Paul, J.—(1966) In " The Biology of Cancer " Edited by E. J. Ambrose and F. J. C. Roe. London (D. Van Nostrand & Co. Ltd.) p. 52.
Potter, V. R.—(1964) *Cancer Res.*, **24**, 1085.
Sanford, K. K., Merwin, R. M., Hobbs, G. L. and Earle, W. R.—(1959) *J. natn. Cancer Inst.*, **23**, 1061.
Sato, H., Belkin, M. and Essner, E.—(1956) *J. natn. Cancer Inst.*, **17**, 1.
Shelton, E. and Rice, M. E.—(1958) *J. natn. Cancer Inst.*, **21**, 137.
Smith, H.—(1958) *A. Rev. Microbiol.*, **12**, 77.—(1964) *Symp. Soc. gen. Microbiol.*, **14**, 1.
Southam, C. M.—(1960) *Cancer Res.*, **20**, 271.
Steel, G. G., Adams, K. and Barrett, J. C.—(1966) *Br. J. Cancer*, **20**, 784.
Takahashi, M.—(1963) *Gann*, **54**, 295.